# Artificial Intelligence, Heuristic Biases, and the Optimization of Health Outcomes: Cautionary Optimism

**DOI:** 10.3390/jcm10225284

**Published:** 2021-11-14

**Authors:** Michael Feehan, Leah A. Owen, Ian M. McKinnon, Margaret M. DeAngelis

**Affiliations:** 1 Cerner Enviza, Kansas City, MO 64117, USA; ian.mckinnon@cernerenviza.com; 2 Department of Population Health Sciences, University of Utah School of Medicine, Salt Lake City, UT 84132, USA; leah.owen@hsc.utah.edu; 3 Department of Ophthalmology, Ross Eye Institute, Jacobs School of Medicine and Biomedical Sciences, State University of New York at Buffalo, Buffalo, NY 14203, USA; 4 Department of Ophthalmology and Visual Sciences, University of Utah School of Medicine, Salt Lake City, UT 84132, USA; 5 Department of Obstetrics and Gynecology, University of Utah School of Medicine, Salt Lake City, UT 84132, USA; 6 Genetics, Genomics and Bioinformatics Graduate Program and Neuroscience Graduate Program, Jacobs, School of Medicine and Biomedical Sciences, University at Buffalo, Buffalo, NY 14203, USA; 7 Veterans Administration Western New York Healthcare System, Buffalo, NY 14212, USA

**Keywords:** heuristics, bias, artificial intelligence, machine learning, health outcomes, population health, ophthalmology, electronic health record

## Abstract

The use of artificial intelligence (AI) and machine learning (ML) in clinical care offers great promise to improve patient health outcomes and reduce health inequity across patient populations. However, inherent biases in these applications, and the subsequent potential risk of harm can limit current use. Multi-modal workflows designed to minimize these limitations in the development, implementation, and evaluation of ML systems in real-world settings are needed to improve efficacy while reducing bias and the risk of potential harms. Comprehensive consideration of rapidly evolving AI technologies and the inherent risks of bias, the expanding volume and nature of data sources, and the evolving regulatory landscapes, can contribute meaningfully to the development of AI-enhanced clinical decision making and the reduction in health inequity.

## 1. Introduction

### 1.1. Artificial Intelligence (AI) and a Reduction in Health Inequity

The growth of artificial intelligence (AI) in clinical practice is driven in part by commercial interests, with AI-focused companies speeding to introduce machine learning (ML) empowered technologies and decision tools for clinical practice; and the recognition by clinicians and regulators that AI holds promise to ease clinicians’ burden while also reducing health inequity. 

The United Nations (UN) has recognized the potential for AI to help achieve United Nations Sustainable Development Goals [1], establishing the digital platform “AI for Good” [2]. At the same time, the UN poses the challenge of how to ensure social and cultural stereotypes are not replicated in AI programming (such as gender discrimination) [1]. United States governmental agencies actively encourage the use of AI in clinical care. For example, commercial entities are having success with the Food and Drug Administration (FDA) granting marketing approvals for Software as a Medical Device (SaMD), and the Center for Medicare and Medicaid Services (CMS) recently awarded a prize to reward “explainable artificial intelligence solutions to help front-line clinicians understand and trust AI-driven data feedback” [3]. This growth in AI implementation will also be enhanced by the National Institutes of Health’s (NIH) Digital Health Equity, Training and Research Consortium. The consortium will address major challenges and barriers of leveraging AI and ML to fuel biomedical innovation through the analyses of medical datasets. A key principle of this effort is to “start by addressing the needs of marginalized communities. If these communities remain an afterthought, the same inequities will be repeated. Address the significant errors, gaps, and racial and gender inequities in EHR data. Using problematic data for models will amplify the gaps.” [4]

### 1.2. Heuristics Can Shape Biases in Clinical Data and ML Predictions

As noted by the UN, the promise of AI is mitigated by the recognition that data and predictive analytics through ML are inherently biased. Patients, caregivers, health care providers (HCPs), reimbursement decision makers, health systems, and regulatory bodies make decisions routinely that impact individuals and the population’s health. In making such decisions, all human actors rely to some degree on “heuristics” [5]. Heuristics are, in part, based on ingrained “rules of thumb” baked into the psyche associated with a stronger probability of behavioral action. A critical consideration in health care decision making, is that any reliance on heuristics is inherently biased [5]. For example, a physician evaluating a patient may make treatment recommendations influenced by mental heuristic “short-cuts” regarding observable or perceived patient characteristics that may be inherently biased and not directly relevant to that patient’s care (e.g., sex and ethnicity). Heuristic bias may exist in more hidden forms, for example, data within health records and case notes may be biased against certain subgroups of patients in ways not obvious to ascertain (e.g., missing data or misclassification). Plous describes the distinction between actuarial predictions (those based on a given set of variables and an outcome) and clinical predictions (predictions based on the judgement of human beings) and concludes that “contrary to common sense, predictions are usually more accurate when they are *not* made by a human decision-maker-even when the decision-maker has full access to the actuarial information” [6] (p. 118). 

Given this humanistic bias, there is natural enthusiasm for the role of artificial intelligence (AI) and machine learning (ML) in informing or even making health care decisions to improve healthcare access, quality, and cost. This may ultimately reduce disparities in population health as these decision-making systems will be optimized through AI to maximize patient health outcomes through optimal treatment pathways and reduce the prevalence of adverse outcomes (e.g., reduction in misdiagnoses, medication errors, etc.) while minimizing unnecessary healthcare expenditure. 

Implicit is the expectancy that the use of ML algorithms in clinical decision support systems [7] will reduce health inequities. On the other hand, Gianfrancesco et al. [8] highlight that ML can output decision rules that may be biased due to non-random missing data or inadequate sample sizes (e.g., on constructs such as ethnicity, gender, and/or socio-economic status), and misclassification and measurement error with practitioner biases impacting the quality of the data themselves. For example, Pierson et al. [9] recently described the potential for supervised ML algorithms to reduce health disparities in pain management using training set data derived from a diverse population (racial, ethnic, socioeconomic, and education). This approach was superior to a reliance on an original severity measure developed “decades ago in white British populations”. It was estimated that the new AI-enhanced criteria would double the eligibility for arthroplasty among Black patients.

## 2. Algorithmic Biases—The Ghost in the Machine?

ML algorithms are themselves subject to biases that flow (a) from the inherent heuristics employed in the act of programming, and (b) in unsupervised learning and reinforcement learning ML models, the biases that are exacerbated by the flaws inherent in the data (which can come from human observers’ errors and miscoding).

### 2.1. Supervised, Unsupervised and Reinforcement Learning

The applications of AI in health care are accelerating through the evolution of ML away from supervised learning to unsupervised and reinforcement learning. In supervised learning, algorithms are programmed by humans as a series of a priori instructions with an expected outcome in mind. For example, a predictive model such as a diagnostic algorithm for age-related macular degeneration (AMD) progression, can be built leveraging a training set of imaging data in which the disease state is coded or labelled by retinal specialists. The algorithms can then evaluate new data that is not in the training set and deliver output/decision rules for improved health outcomes such as flags in the system to identify patients at higher risk of AMD progression. Humans determine the rules about which data are to be included, the criteria used to ascertain the risk characteristics of each image, and the outcome thresholds that will define how a patient “at risk” is identified. Heuristic biases can intrude in those decisions, as O’Neill points out [10] (p. 21), a “model’s blind spots reflect the judgements and priorities of its creators”. 

In contrast, using unsupervised learning, the algorithms operate without human-proved data labels for the training set and can uncover relationships in the data that perhaps may be “new” and not readily known to examining physicians, especially if data outside the image data per se are included in that training. Furthermore, reinforcement learning can be applied whereby algorithms use a positive reinforcement reward system building predictions from the data available in a dataset from repeated simulations, with a goal to achieve an a priori optimal outcome (e.g., minimization of false positive and false negatives in diagnostic risk ascertainment for AMD progression).

In supervised learning approaches, any heuristic biases of the human’s involved can influence the AI through the choice of training set data and accurate coding of data in the training set, especially when coding requires the subjective evaluation of the patient or interpretation of laboratory, imaging, and other data. However, in an unsupervised approach, ML algorithms may also be biased in ways *unfathomable* to the observer. Models developed like this can be inherently uninterpretable by human agents as ML does not output how it arrived at the predictions. 

Biases in ML algorithms for clinical decision support tools can contribute to socioeconomic inequity in public health, especially in unsupervised scenarios when the machine may be at liberty to determine which variables to include in a model and the weight given to each without human guidance. This is particularly true when the datasets extracted from the EHR are becoming vastly more complex in terms of breadth of sources and volume of data available (laboratory results, genomic mapping, case notes, wearables, telehealth sensors, etc.). ML algorithms may encode the biases of their human creators, exacerbated by its scale if applied around the world, and if they overly rely on uninterpretable models [11].

### 2.2. Ophthalmology Imaging—A Case in Point

The pace of AI and ML development is accelerating, and applications across multiple disease states are burgeoning. This is strikingly so in ophthalmology given the field’s increasing reliance on innovative enhanced imaging technologies for age-related macular degeneration [12,13,14,15,16], diabetic retinopathy [17,18,19,20,21], and glaucoma [22,23,24,25,26] in clinical practice. It is also an emerging technology for large-scale screening for vision impairment [27,28] and an aid for diseases in pediatric populations when there can be significant subjectivity and variation in diagnostic agreement [29,30].

In a recent review of ML in ophthalmology, Balyen and Peto [31] highlight the value of such “unmanned” imaging algorithms given the time-consuming nature of image review by physicians, its cost, the risk of making human errors, and the unavailability of retinal specialists in certain countries. Such algorithms have been shown to have high accuracy, sensitivity, and specificity for the detection and grading of diabetic retinopathy, AMD, and glaucoma. However, the authors acknowledge the biases that can occur with human graders as conventional diagnostic methods for the eye rely on the experience and knowledge of the physician, and that over-reliance can lead to high rates of misdiagnosis. 

## 3. Success in the Lab May Not Translate to the Real-World Clinic

Success in the laboratory setting does not always equate with success in real-world settings. This lack of generalization is a form of bias that can be particularly acute when the predictive model is built using training data that are narrow and incompletely reflect the variability present in the real-world setting, or the algorithms take heuristic shortcuts that work in one setting but are not generalizable to others. This is analogous to reduced drug efficacy in the general population as compared with initial clinical trial data. This is widely recognized to result from clinical trial patient homogeneity not being reflective of a heterogeneous population.

### Underspecification and Shortcut Learning

Computer scientists may attribute this to underspecification in model development. In these situations, the solution to a problem is underspecified if there are many distinct solutions that can solve the problem equally well [32]. Geirhos et al. [33] use the term “shortcut learning” when describing failings in deep neural networks when ML algorithms are applied in the real world. They cite a study showing an ML system could detect pneumonia from X-rays from certain hospitals but was less accurate for other hospitals [34]. Behind the scenes, the algorithm was including hospital identification tags in its learning with associated hospital-specific pneumonia prevalence data, which then failed to transfer to novel hospitals. The algorithms were essentially taking a heuristic shortcut to calibrate predictions, using an approach “to solve the problem differently than intended” by its creators [34].

D’Amour et al. [32] examined an ML model trained to detect diabetic macular edema (DME) and diabetic retinopathy (DR) within retinal fundus images. A goal of this application would be to help reduce health disparities in blinding disease, applying such algorithms across populations where “clinical expertise may be spread thin”. However the models pre-trained on fundus images from EyePACS in the USA and other hospitals in India [35] worked well, but not so well when data from camera types and settings not used in the training set were included. Similarly, vision impairment screening algorithms trained on Asian images performed less well on a US dataset of eyes, reinforcing the need for diverse training sets across populations [27].

## 4. What Is in The Black Box?

A key driver of the use of ML in healthcare is to uncover novel structures within data that may not be specified a priori by the research or clinical teams. This is especially so when large volumes of data are freely available in an EHR. One challenging phenomenon is that the methods by which ML algorithms provide output are often not well understood. Even in high-stakes decision making such as health care, “black box” machine models exist, which do not explain their predictions in ways that humans can understand [36], and, as Zech et al. note, may be solving problems using data and relationships between data in ways unintended by the creators [34]. Models that can be *interpretable* by humans are more likely to be sparse and transparent, but this conflicts with the possibility that clinicians and researchers may also follow an underlying heuristic that “more data is better”, seeking to add additional layers of complexity from which the AI may uncover novel truths. As noted below, this lack of interpretability is a significant drawback for regulatory approval.

## 5. Hearing the Patient and Providers Voices—Leverage Unstructured Case Note Data

Algorithmic sensitivity and specificity in ophthalmic image-based diagnoses [11] have been well studied, but the inclusion of broader health data within the EHR to guide treatment and minimize inequity in health care delivery is less common. Researchers tend to focus on the structured imaging data available in the EHR, and do not often address the wealth of unstructured data in the clinical record that can be extracted through natural language processing (NLP) and incorporated in predictive ML algorithms and decision tools. 

Our research has demonstrated high levels of medication non-adherence in general, and for asthma and glaucoma [37,38,39]. Since the appropriate use of glaucoma eye drops can drastically reduce the risk of irreversible blindness, we see great promise in using ML algorithms to identify patients at risk of non-adherence using the breadth of data available in the EHR. In this case we intend to explore this with the Veterans Health Information Systems and Technology Architecture (VISTA). These data may include imaging and visual acuity data, but also laboratory measures, the medication burden for patients with multiple comorbidities, and data likely to reflect social determinants of health (at the patient, practitioner, and clinical setting level). A key aspect of this approach will be to use NLP and ML algorithms to identify, quantify, and characterize the non-adherent population when adherence data per se may not be explicitly coded in the record. 

NLP has been used with unstructured VISTA data to enhance screening for military sexual trauma (MST). The overall estimate of MST in women and men increased from 8.1% to 13.1% when NLP was applied [40]. In another EHR system, NLP has recently been shown to achieve high specificity for identifying patients with pulmonary embolism events (PE) and greater specificity than ICD-10 coding in the EHR [41]. A challenge here is that bias in ML models can be exacerbated in NLP. D’Amour et al. cite examples of bias through stereotypical gendered associations in text, where models “learn” spurious correlations and shortcuts [32] and can lead to a “severe lack of robustness” and inability to generalize to more challenging test conditions [34]. 

## 6. Optimization of Clinical Trials with AI

The US FDA recognizes that the under-representation of certain populations in clinical trials is problematic, and it encourages more participation and diversity in clinical trials, focusing largely on ethnicity or race, sex/gender, and age. Inadequate diversity within trial populations can lead to inequity in the risk/benefit assessment for under-represented groups. 

Harrer et al. [42] suggest that AI can positively contribute to clinical trial design and execution by leveraging data in the EHR, medical literature, and trial databases to improve patient–trial matching and recruitment. AI can also be used to monitor patients continuously over the course of trials (e.g., adherence to study investigational products, and the prediction and minimization of study dropout).

Noting the success of the collaboration between the Moorfields Eye Hospital and Alphabet’s DeepMind Health, which showed that AI tools outperformed multiple experts in interpreting optical coherence tomography scans [43], Ahuja and Halperin [44] recommended such approaches should be subjected to rigorous randomized control trials and gain regulatory approval. We have identified genetic factors differentiating subtypes of patients with AMD [45]. A potential clinical trial in this area could leverage ML algorithms to electronically genotype and phenotype patients, analyzing genetic data, fundus and other imagery, and the host of sociodemographic and comorbidity data available across the EHR to identify a large pool of potential candidate subjects for trials. This is particularly true when large EHR providers have data sharing agreements in place across multiple provider organizations, and the breadth of data can be deep and varied across populations. This can be a powerful approach when identifying low-prevalence populations of patients with rare diseases, or unique populations such as those with pediatric retinopathy immaturity noted above [30]. 

Such matching of identified candidate subjects to trials through AI has been successfully demonstrated with structured and unstructured EHR data (including genetic markers and histology) for breast and lung cancer patients [46]. A recent cardiovascular randomized clinical trial also demonstrated that an AI-powered clinical decision support tool can enable the early diagnosis of low ejection fraction in routine primary care [47].

When AI is used in any capacity in clinical trials, it also should be reported appropriately. Campbell et al. [11] provide a recent update on the evolving standards and recommendations for AI in both design and protocol development.

## 7. Regulatory Considerations

Given the significant potential to improve patient outcomes through AI, there is a real need to ensure that the pace of development does not outstrip regulatory oversight.

### 7.1. GDPR, ICMR, and the European Experience

In the European Union, the General Data Protection Regulation (GDPR) has recognized the importance of transparency in AI-based decision making. Implicit in the GDPR is the right to non-discrimination, but, as Goodman and Flaxman note, any use of AI and algorithmic processing that may profile people for resource allocation is inherently discriminatory [48]. In the GDPR Section 22 [49] it expressly states: “The data subject shall have the right not to be subject to a decision based solely on automated processing, including profiling, which produces legal effects concerning him or her or similarly significantly affects him or her.” In the GDPR there is an implicit expectancy that decisions for a patient classification must be dependent on that clinician or health system’s ability to satisfactorily *explain* how the ML model arrives at that patient’s classification.

In August 2021, the International Coalition of Medicines Regulatory Authorities (ICMR) published recommendations to assist regulators in considering the challenges of AI in medicine regulation [50]. They noted that regulators need to elaborate on a risk-based assessment of AI, expressly taking into consideration a “sufficient level of understandability and regulatory access to the employed algorithms and underlying datasets” while acknowledging “limits to validation and predictability may have to be identified and tolerated when, for example, the AI is to learn, adapt or evolve autonomously” and the later should be considered “higher-risk” uses. They conclude that to address the unpredictable and opaque nature of AI updates, the post-authorization management of medicines may need to be adopted to accommodate updates to AI associated with a medicinal product.

### 7.2. FDA and the US Experience

According to STAT [51], the Food and Drug Administration (FDA) approved 161 AI products between 2012 and 2020, yet only 73 disclosed in public documents the amount of patient data used to validate the performance of their devices. Of those, only seven reported the racial makeup of their study populations and only 13 reported a gender breakdown. STAT cited Kayte Spector-Baghdady, a lawyer and professor of bioethics at the University of Michigan, saying “I do think there is a lot of promise to AI, but if we’re not even requesting that the manufacturers be transparent about their reference datasets at submission, how are people going to know the limitations of that AI instrument?” 

The FDA categorizes products that rely on AI/ML as Software as a Medical Device (SaMD) [52]. In January 2021, the FDA released an action plan [53] for SaMD, highlighting the need for a Pre-Determined Change Control Plan, with the expectancy of “transparency and real-world performance monitoring by manufacturers that could enable FDA and manufacturers to evaluate and monitor a software product from its premarket development through postmarket performance.” As with FDA Good Clinical Practice guidelines for investigators involved in clinical trials, the FDA recommends the adoption of Good Machine Learning Practice (GMLP) [54]. The action plan highlights the need for patient-centricity in driving development and implementation with a focus on transparency to patients and health care providers of the risks and benefits of AI/ML-based medical devices, “which may learn and change over time, and which may incorporate algorithms exhibiting a degree of opacity.” The FDA’s action plan specifically addresses the possible risk of algorithmic biases, and its commitment to support regulatory science efforts to develop methods to evaluate algorithms for the identification and elimination of such bias. 

## 8. Conclusions and Recommendations

As public health consultants and clinical researchers we remind ourselves of the importance of “reflective practice”, and perhaps encourage the need for all to pause and reflect on sources of bias when thinking of designing or implementing clinical tools relying on AI and ML. Explicit consideration of sources of bias may improve the accuracy of these tools, aid their transition from the computer science lab to the clinic, overcome any practitioner resistance to their use, improve the design of clinical trials, and, ultimately, enhance the likelihood of regulatory approval to improve public health.

- Expressly and consciously appreciate the presence of heuristically formed biases in the minds of the researchers and clinicians designing the work, and the NLP and ML computer scientists and their programming;
- Closely evaluate the data selected for use in training ML algorithms and any bias in that selection, along with potential biases inherent in the EHR data to which that ML algorithm will be applied;
- When setting a training set, consider the inclusion of variables outside the “core” clinical data of immediate interest, particularly data on factors indicative of the social determinants of health and health inequity (and the biases in the presence/absence of those data, and in its coding/entry into the EHR);
- Explore the possibility of using NLP to expand the breadth of data in ML models, given the potential for unstructured data in the EHR case notes to reveal drivers of positive/adverse health outcomes. If so, carefully consider the biases inherent on what may (or may not) be entered into a free-text field in the record, and how it is described by the writer;
- Build probes and outputs into any system to ensure that the ML models are interpretable and understandable by the human team. With large heterogenous datasets and the use of unsupervised ML, this can be a challenging task;
- Design the system to ensure compliance with relevant regulatory bodies in the country of development, and the countries where the technology may be employed, recognizing the evolving landscape of regulation in this space and the rights of patients for transparency in decision making. Be able to defend precisely how the ML system classified patients;
- Consider the end use of the application and the sources of potential underspecification of the ML models that could result in a failure to translate to real-world patient outcomes;
- In designing clinical studies and their protocols, ensure any ML used to enhance subject/patient participation does not create additional bias in eligibility, and at study close ensure compliance with the rapidly evolving standards for reporting around the use of AI in clinical research;
- Anticipate the need to consider a Pre-Determined Change Control Plan at the outset for ongoing adjustment of ML models, especially when those models may be applied to new populations outside the population that generated the training set;
- Proactively design for the real-world evaluation of the application post-authorization, positing how its use may be monitored for both efficacy and safety outcomes.

We are in an exciting era where AI and ML can potentially transform the clinical care and healthcare landscape, reduce inequity in health outcomes, and improve the treatment lifecycle from development to postmarket evaluation. However, let us ensure we consider the heuristic biases inherent in all levels of these systems as we move forward into the future.

## Data Availability

Not applicable.
